# Microbial metabolite p-cresol inhibits gut hormone expression and regulates small intestinal transit in mice

**DOI:** 10.3389/fendo.2023.1200391

**Published:** 2023-07-18

**Authors:** Pernille Baumann Toft, Amanda Marie Vanslette, Kajetan Trošt, Thomas Moritz, Matthew Paul Gillum, Fredrik Bäckhed, Tulika Arora

**Affiliations:** ^1^ Novo Nordisk Foundation Center for Basic Metabolic Research, Faculty of Health and Medical Sciences, University of Copenhagen, Copenhagen, Denmark; ^2^ Wallenberg Laboratory, Department of Molecular and Clinical Medicine, University of Gothenburg, Gothenburg, Sweden; ^3^ Department of Clinical Physiology, Region Västra Götaland, Sahlgrenska University Hospital, Gothenburg, Sweden

**Keywords:** p-cresol, GLP-1, small intestinal transit, microbial metabolite, metabolic disease

## Abstract

p-cresol is a metabolite produced by microbial metabolism of aromatic amino acid tyrosine. p-cresol and its conjugated forms, p-cresyl sulfate and p-cresyl glucuronide, are uremic toxins that correlate positively with chronic kidney disease and diabetes pathogenesis. However, how p-cresol affects gut hormones is unclear. Here, we expose immortalized GLUTag cells to increasing concentrations of p-cresol and found that p-cresol inhibited *Gcg* expression and reduced glucagon-like peptide-1 (GLP-1) secretion *in vitro*. In mice, administration of p-cresol in the drinking water for 2 weeks reduced the transcript levels of *Gcg* and other gut hormones in the colon; however, it did not affect either fasting or glucose-induced plasma GLP-1 levels. Furthermore, it did not affect glucose tolerance but promoted faster small intestinal transit in mice. Overall, our data suggest that microbial metabolite p-cresol suppresses transcript levels of gut hormones and regulates small intestinal transit in mice.

## Introduction

Gut microbiota regulates the production of a plethora of microbial metabolites that are recently revealed to play an important role in the host’s metabolic regulation (1, 2). Fermentable dietary fiber-derived microbial metabolites such as short-chain fatty acids (SCFAs) have been of major interest (3, 4), but there is limited literature on the physiological effects of protein-derived microbial metabolites. p-cresol is produced from microbial putrefaction of aromatic amino acid tyrosine in the colon and is absorbed by colonocytes where it can be further metabolized to p-cresyl sulfate (p-CS) and p-cresyl glucuronide (p-CG) prior to release into the portal vein (5). Unmetabolized p-cresol can also be metabolized to p-CS and p-CG in the liver before entering the systemic circulation. In plasma, p-cresol, p-CS, and p-CG bind to albumin in a reversible manner, whereas free forms of p-cresol and its conjugates are excreted in the urine (6). Plasma and urinary levels of p-cresol and p-CS are elevated in patients with chronic kidney disease (7–9) and type 2 diabetes (10, 11) and positively associate with disease pathogenesis. p-cresol and its conjugates are also elevated in the urine of children with autism (12) and in the serum of patients with Parkinson’s disease (13) or chronic kidney disease-associated cardiovascular complications (11, 14). In addition, protein-derived metabolites produced in the colon, including p-CS, can also act locally and are associated with slower colonic transit in healthy individuals (15).

Enteroendocrine cells (EECs) reside along the length of the intestine and sense fluctuations in dietary and gut microbiota-regulated metabolites (16). Multiple gut microbiota-regulated metabolites such as SCFAs, indole, and secondary bile acids affect the secretion and expression of gut hormones (17). Gut hormones such as glucagon-like peptide-1 (GLP-1), Peptide YY (PYY), and neurotensin (NTS) regulate multiple physiological functions both locally such as intestinal transit and *via* their action on peripheral organs such as the pancreas influencing glucose metabolism (18, 19). It is technically challenging to measure p-cresol concentrations in the gut due to transformation into conjugated counterparts; one report suggests that free p-cresol concentrations can reach concentration as high as 0.9 µmoles/g of large intestinal contents of pigs (20). Therefore, it is conceivable that p-cresol might regulate the expression and secretion of gut hormones, which is currently unclear.

To this end, we used both *in vitro* and *in vivo* procedures to unravel the effects of p-cresol on gut hormone expression and secretion. We found that p-cresol inhibits proglucagon (*Gcg)* expression *in vitro* and that p-cresol administration to mice reduced the RNA expression of *Gcg* and other gut hormones in the colon and decreased small intestinal transit time in mice.

## Methods

### *In vitro* procedures

GLUTag cells were seeded at the density of 100,000 cells per well in poly-D-lysine-coated 24-well plates. All experiments were performed 24–48 h after seeding when cells reached 70%–80% confluence. For the expression assay, cells were washed once with Dulbecco’s Modified Eagle’s Medium (DMEM) containing 1% fetal bovine serum (FBS) and 0.2% bovine serum albumin (BSA) and incubated with different concentrations of p-cresol (10, 100, and 1,000 µM) for 24 h. The cells were scraped and collected in RLT lysis buffer containing 10 µl β-mercaptoethanol/ml. The lysates were snap-frozen until RNA extraction.

For the secretion assay, the cells were washed three times with secretion buffer (containing 4.5 mM KCl, 138 mM NaCl, 4.2 mM NaHCO_3_, 1.2 mM NaH_2_PO_4_, 2.6 mM CaCl_2_, 1.2 mM MgCl_2_, and 10 mM HEPES, pH 7.4) containing 0.1% BSA. The cells were then incubated with different concentrations of p-cresol (10, 100, and 1,000 µM) with or without positive control, 10 µM chemical TGR5 agonist MerckV, for 2 h. Following incubation, secretion buffer was collected and centrifuged at 1,500×g to remove any floating cells. Finally, supernatants were collected and snap-frozen until GLP-1 analysis. All secretion and expression assays were repeated independently at least three times, and statistics was performed on the average of data points per independent experiment.

### Mouse studies

The mouse experiments were performed in accordance with the bioethical guidelines, which are fully compliant with institutional (University of Copenhagen) accepted principles for the care and use of laboratory animals approved by the Animal Experiments Inspectorate under the Danish Ministry of Food, Agriculture and Fisheries. Swiss Webster males (n = 6–7) were group-housed in individual ventilated cages at a temperature- and humidity-controlled room at 22°C with 12:12 light/dark cycle. Mice were fed a Western-style diet (Formula D12331, Research Diets) and then randomly allocated to receive either drinking water or water supplemented with 0.25 g/L p-cresol (12) (W233706, Sigma-Aldrich^®^) for 2 weeks. Water bottles containing p-cresol were changed twice weekly. Body weight, food and water intake were monitored two times a week. At the end of the experiment, blood samples were collected by puncturing the retro-orbital sinus and plasma was separated by centrifugation at 10,000×g for 5 min. Liver, ileum, cecum, and proximal colon were collected and snap-frozen. In a separate cohort of female mice (n = 4–5), following 2 weeks of p-cresol supplementation, an oral glucose tolerance test was performed by administering 30% glucose solution (2 g/kg body weight) to 4-h fasted mice and blood samples were collected from the retro-orbital sinus at baseline and 10 min following glucose gavage. Glucose readings were taken before and 10, 30, 60, 90, and 120 min after the oral glucose gavage. Three days later, 4-h fasted mice were orally gavaged with 200 µl of 6% carmine red solution prepared in 0.5% methylcellulose to assess small intestinal transit. After 45 min, whole small intestinal tract was removed and a visible proportion (by eye) of small intestine in which carmine red has traveled was expressed as the percentage of total small intestinal length to determine small intestinal transit as described before (21).

### RNA extraction and cDNA synthesis

RNA was extracted from cell lysates and intestinal segments following kit instructions (RNAeasy Mini kit, Qiagen). cDNA was synthesized using the SuperScriptTM III Reverse Transcriptase (ThermoFisher Scientific). Gene expression was analyzed in a qPCR reaction containing 5 μl PrecisionPLUS 2× qPCR Master Mix (Primer design), 0.5 μl forward/reverse primer (10 μM), and 2.5 μl sterile water per reaction. For cDNA from GLUTag cell experiments, the housekeeping gene *Gapdh* (glyceraldehyde-3-phosphate dehydrogenase) was used as endogenous control, whereas for cDNA from mouse tissue, the housekeeping gene *Ywhaz* (tyrosine 3-monooxygenase/tryptophan 5-monooxygenase activation protein zeta) was used as endogenous control. Relative expression was calculated using 2^-ΔΔCt^ method. Forward and reverse primer sequences are as follows: *Gapdh* 5´-AGGGCTCATGACCACAGTC-3´, 5´-GGATGCAGGGATGATGTTCT-3´; *Ywhaz* 5’-AGACGGAAGGTGCTGAGAAA-3’, 5’-GAAGCATTGGGGATCAAGAA-3’; *Gcg* 5´-GCTTATAATGCTGGTGCAAG-3´, 5´-TTCATCTCATCAGGGTCCTC-3´; *Pyy* 5´-GCAGCGGTATGGAAAAAGAG-3´, 5´-GTCGCTGTCGTCTGTGAAGA-3´; *Nts* 5´-CTGGTGTGCCTGACTCTCCT-3´, 5´-TCACATCTTCTTCTGAATCTGAGC-3´; *Cck* 5´-CCCCAATGTGAAATCTGTCC-3´, 5´-GGTCTGGGAGTCACTGAAGG-3´.

### GLP-1 measurement

Total GLP-1 was analyzed in the secretion buffer from GLUTag cells and plasma samples from mice using V-plex GLP-1 Total kit on the Mesoscale Discovery platform.

### LC-MS measurement of p-cresol conjugates

#### Sample preparation

Method applied was previously reported (22) and was modified to fit specific metabolites and matrixes used. Sample preparation was divided into two parts. The same protocol was used for cecal and liver tissue samples and another for plasma samples.

In the first step, tissue samples were cryo-pulverized using CPO2 (Covaris, USA). Deep frozen tissue was inserted into cryo-bag (TT05M XT 520140, Covaris, USA) and pulverized. Powder was stored on -80°C until further processing. Water was removed from the samples with ScanVac CoolSafe freeze drier (Labogene, Denmark). Samples were dried for 20 h, and weight was measured. Proteins were removed using organic solvent extraction. Dried tissue powder was mixed with 180 μl of methanol containing 0.1 mg/l of internal standard 3-hydroxy tridecanoic acid and left for 30 min to precipitate proteins on ice. After that, extracts were centrifuged on 4°C and 10,000 rpm for 3 min (5810R, Eppendorf, Germany). Then, 120 μl of supernatant was collected and dried under a stream of nitrogen and resuspended in 60 μl of methanol:water = 1:1 (v:v).

A similar procedure was applied for plasma metabolite extraction. Here, 30 µl of plasma was mixed with 180 μl of methanol containing 0.1 mg/l of 3-hydroxy tridecanoic acid. After protein precipitation and centrifugation with the same conditions as tissue samples, 120 µl of supernatant was taken and resolved in 60 µl of methanol:water = 1:1 (v:v). Peak areas were normalized to the internal standard.

### Instrumental analysis

The system used was composed of high-performance liquid chromatograph (1290 Infinity II, Agilent, USA) and mass spectrometer (TimsTof Pro, Bruker, Germany). Metabolite separation was achieved with reversed-phase column (TSS T3 10 cm × 2.1 × 1.8 μm, Waters, USA) and with the gradient of mobile phases. Mobile phase A was water with 0.1% of formic acid, while mobile phase B consisted of acetonitrile:isopropanol = 3:1 (v:v) with added 0.1 % of formic acid. Chromatographic gradient started with 3% of mobile phase B, which was increased to 100% in 9 min where it was kept stable for 5 min. After that system was reequilibrated to initial conditions, column temperature was kept on 40°C and injection volume was 10 μl.

Acquisition of mass spectra was set to profiling mode, ranging from 50 to 500 Dalton, and acquisition speed was set to 2 Hz. Spectra were acquired in negative ionization mode. A similar method with automatic acquisition of fragmentation spectra was applied for metabolite identification. Due to the lack of metabolite standards, metabolites were identified using exact mass and fragmentation spectra. Peak area of metabolites were extracted using MzMine 2.53 in targeted mode, and metabolites were normalized to internal standard and to the tissue weight.

### Statistical analysis

All statistical analyses were performed using GraphPad Prism 9.4.0 and presented as mean ± standard error of the mean (SEM). In cell experiments, nonparametric Kruskal–Wallis test followed by Dunn’s test was used to assess the significance between the treatments. Unpaired nonparametric Mann–Whitney U test was used to determine the significance between control and p-cresol-supplemented groups in the mouse experiments. Significance was established at p < 0.05.

## Results

### p-cresol Inhibits *Gcg* expression and chemically induced GLP-1 secretion *in vitro*

**Figure 1:**
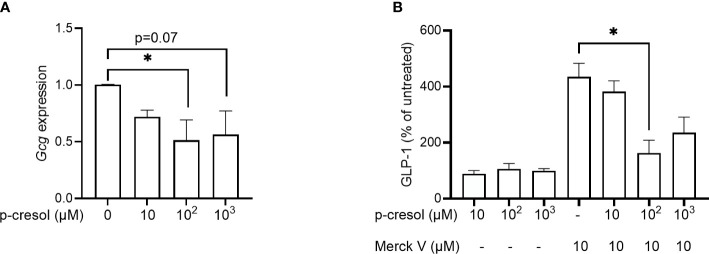
**(A)** Expression of proglucagon (*Gcg*) in GLUTag cells exposed to different concentrations of p-cresol and **(B)** GLP-1 secretion in GLUTag cells exposed to different concentrations of p-cresol in the absence and presence of 10 µM chemical TGR5 agonist MerckV. Each bar represents the mean of three independent measurements. Data were analyzed using nonparametric Kruskal–Wallis test followed by Dunn’s *post-hoc* analysis * p < 0.05.

To investigate if p-cresol affects GLP-1 expression and secretion, we first exposed GLUTag cells to increasing concentrations of p-cresol to assess gene expression and secretion of the peptide. To assess the expression, we exposed GLUTag cells with increasing concentrations of p-cresol for 24 h and observed that p-cresol inhibited proglucagon (*Gcg)* expression at 100- (p = 0.04) and 1,000 µM concentrations (p = 0.07) (**Figure 1A**).

In the secretion assay, where GLUTag cells were exposed to increasing concentrations of p-cresol for 2 h, we found that p-cresol itself neither stimulated nor inhibited GLP-1 secretion at any concentration (**Figure 1B**). Next, we used increasing concentrations of p-cresol along with chemical TGR5 agonist MerckV (23). As expected, MerckV induced GLP-1 secretion 4-fold, which was significantly reduced to half in the presence of 100 µM p-cresol (**Figure 1B**). A similar reduction was observed with 1,000 µM p-cresol; however, it remained nonsignificant (**Figure 1B**). Thus, p-cresol inhibits *Gcg* expression and TGR5-induced GLP-1 secretion.

### p-cresol inhibits *Gcg* expression and regulates small intestinal transit *in vivo*

**Figure 2:**
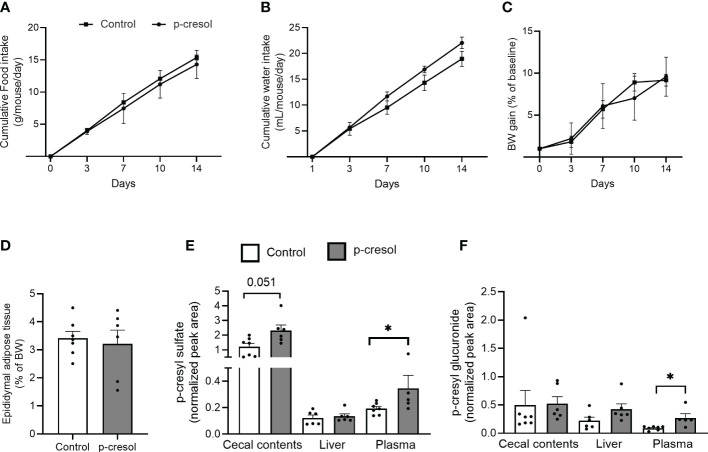
Cumulative **(A)** food intake and **(B)** water intake, **(C)** body weight gain, **(D)** epididymal adipose tissue mass (expressed as the percentage of body weight) in control and p-cresol-supplemented mice (n = 6–7). **(E)** p-cresyl sulfate and **(F)** p-cresyl glucuronide (expressed as normalized peak area) in cecal contents, liver, and plasma of control and p-cresol-supplemented mice (n = 6–7). Data were analyzed using nonparametric Mann–Whitney U test, * p < 0.05.

To validate our *in vitro* findings *in vivo*, we supplemented drinking water of mice with p-cresol for 2 weeks. Despite the aromatic nature of p-cresol, we found no significant effect of p-cresol on food or water intake of mice (**Figures 2A, B**), indicating that the dose was well tolerated. This is in accordance with a previous study (12) where p-cresol was administered at the same dose for more than 4 weeks and did not affect food and water intake. In our study, body weight gain (**Figure 2C**) and epididymal adipose tissue mass (**Figure 2D**) of the mice did not differ between the groups. In contrast, previous reports demonstrated that intraperitoneal administration of p-cresol (24) or p-CS (25) reduced the epididymal adipose tissue mass and promoted the mobilization of fat content from the epididymal depot to the liver and muscle in mice.

p-cresol is metabolized to p-CS and p-CG in the intestine and liver. Therefore, we measured concentrations of free p-cresol and both conjugated forms of p-cresol in the plasma, liver, and cecal contents. We could unfortunately not detect free form of p-cresol using our method; however, we could measure both p-CS and p-CG in all of the three tissue compartments. p-CS, which is the major detoxified conjugated form of p-cresol, was higher in both plasma and cecal contents (**Figure 2E**), while p-CG was higher in the plasma of the p-cresol-supplemented group compared with control (**Figure 2F**).

**Figure 3:**
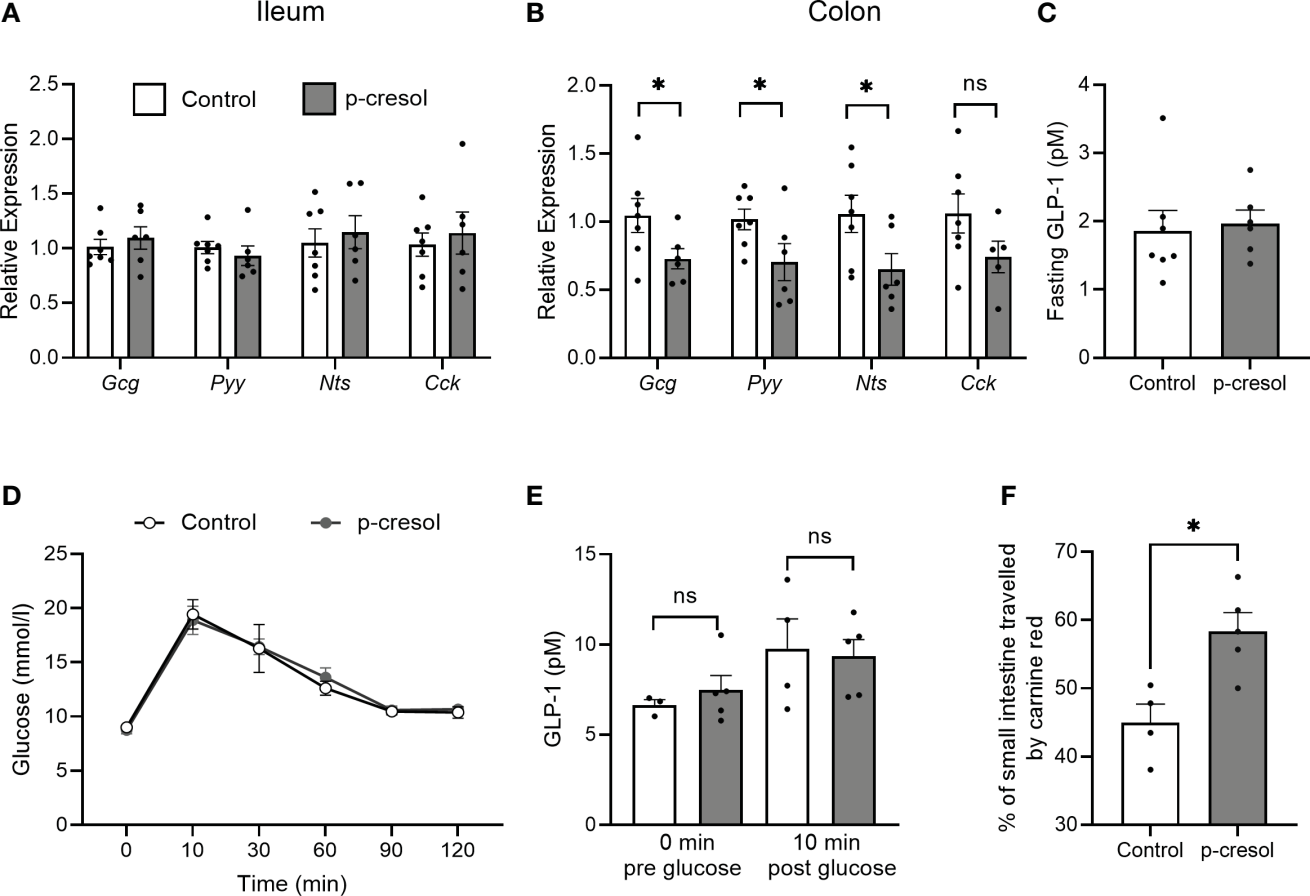
RNA expression of proglucagon (*Gcg*), peptide YY (*Pyy*), neurotensin (*Nts*), and cholecystokinin (*Cck*) in **(A)** ileum **(B)** and proximal colon and **(C)** fasting plasma GLP-1 in control and p-cresol-supplemented mice (n = 6–7). **(D)** Oral glucose tolerance test, **(E)** GLP-1 pre- and post-glucose gavage, and **(F)** small intestinal transit (expressed as the percentage of the small intestine traveled by carmine red relative to total small intestinal length) in control and p-cresol-supplemented mice (n = 4–5). Data were analyzed using nonparametric Mann–Whitney U test, * p < 0.05. ns, not significant.

Next, we measured the RNA expression of *Gcg* and other gut hormones in ileum and colonic sections. In ileum, there was no difference in *Gcg* or other gut hormone expression (**Figure 3A**), whereas colonic *Gcg* transcript levels were significantly reduced in p-cresol-supplemented group compared with control (**Figure 3B**). Similarly, the RNA expression of other gut hormones such as *Pyy* and *Nts* showed the same trend and was significantly reduced when mice were exposed to p-cresol in the water (**Figure 3B**). However, we did not observe a difference in fasting plasma GLP-1 levels between the groups (**Figure 3C**).

Since our *in vitro* results also indicate that p-cresol did not affect GLP-1 secretion on its own but only inhibits the GLP-1 secretion in a stimulated state, we performed an oral glucose tolerance test in a separate cohort of mice and measured GLP-1 in plasma samples collected before and 10 min after glucose gavage. In response to the oral glucose challenge, neither blood glucose levels (**Figure 3D**) nor GLP-1 levels differed between the groups (**Figure 3E**). GLP-1 and other gut hormones play an important role in the regulation of small intestinal transit; therefore, we measured small intestinal transit time in both groups of mice and found that p-cresol-supplemented mice displayed a faster small intestinal transit compared with that of controls (**Figure 3F**). Overall, using *in vitro* and *in vivo* procedures, our data suggest that microbial metabolite p-cresol, which is a known uremic toxin and is associated with diabetes phenotype, reduces the transcription of gut hormones and regulates small intestinal transit *in vivo*.

## Discussion

With advances in gut microbiome research, gut microbiota-regulated microbial metabolites are recognized as intermediary molecules connecting the gut to the peripheral organs. Microbial metabolites act as chemical messengers, energy source, or signaling molecules (2). Of the macromolecules in the diet, dietary fibers and their fermentable counterpart SCFAs have gained significant attention for their role in the regulation of diverse physiological functions (3, 4) spanning from fuel source for colonocytes (26) to gut hormone regulation (18) and modulation of immune function (27). Recent interest has shifted to identify microbial metabolites derived from other dietary macromolecules, such as fats and proteins. An arsenal of host proteases is produced to digest dietary proteins efficiently in the upper gut. However, the residual peptides and amino acids indeed reach the lower gut and are subjected to microbial putrefaction leading to the production of multiple metabolites (28, 29). Here, we have characterized the functions of aromatic amino acid tyrosine-derived microbial metabolite p-cresol (6) on gut hormone producing EECs and identified that p-cresol inhibits colonic *Gcg* RNA expression and regulates small intestinal transit in mice.

Our *in vitro* analysis on GLUTag cells revealed that p-cresol inhibited *Gcg* expression and GLP-1 secretion in a stimulated state. Using the same cell model, Chimerel et al. (30) demonstrated that another protein-derived microbial metabolite, indole, inhibits glucose-induced GLP-1 secretion in GLUTag cells when exposed for 2 h. Interestingly, neither p-cresol nor indole affected GLP-1 secretion by itself but inhibited agonist (TGR5 agonist or glucose, respectively)-induced secretion, indicating that these metabolites possibly suppress induced hormone release.

Next, our *in vivo* analysis where p-cresol was administered in the drinking water of mice showed a significant reduction in colonic *Gcg* expression but did not affect glucose tolerance. Current literature on the role of p-cresol on glucose metabolism presents contrasting data. One study reported that administration of a nontoxic dose of p-cresol (0.5 mg/kg/day) improved glucose tolerance in mice with marked increase in β-cell proliferation (31). In contrast, using another mouse model where p-cresol was injected intraperitoneally twice daily for 4 weeks promoted hepatic lipotoxicity and worsened sensitivity to insulin (24). However, these studies did not study the effect on gut hormones, and it is pertinent that differences in doses and route of administration may account for different metabolic responses between our study and reported observations.

In addition, we did not observe differences in either fasting or oral glucose gavage-induced increase in plasma GLP-1 levels between control and p-cresol-supplemented groups, indicating that differences in colonic *Gcg* transcript levels perhaps do not correspond to differences in systemic GLP-1 levels, thus resulting in no effect on glucose tolerance in our study. It is consistent with the hypothesis that GLP-1 released from the distal intestine is unlikely involved in the regulation of glucose metabolism (32). However, a recent study using a sophisticated mouse model that enables selective activation of L-cells in the distal gut demonstrated improvement in glucose tolerance (33).

In addition to glucose regulation, GLP-1 (34) and other gut hormones such as PYY (35) have an important role in the regulation of intestinal motility. Intestinal motility is an important factor in shaping gut microbiota composition and eventual production of metabolites (15, 36). We found that p-cresol-supplemented mice showed faster small intestinal transit than control mice probably due to lower colonic RNA expression of *Gcg* and *Pyy* that are known to inhibit small intestinal motility (37). This is consistent with a previous study that suggested slower small intestinal transit in a germ-free mouse model mediated by higher colonic *Gcg* RNA levels that was dependent on GLP-1 receptor (21). In addition, selective activation of L-cells in the gut using intersectional genetics showed marked expression of *Gcg* in the colon and slower small intestinal transit in mice (37). However, transit of food through the small intestine is determined by multiple factors, such as the rate of gastric emptying, diet–microbiota interactions (38), and the enteric nervous system (39); therefore, we cannot exclude that these factors might be at play here that need to be further studied.

In conclusion, here we have identified p-cresol as a metabolite with potential to modulate intestinal transit *via* its effect on gut hormones. A thorough investigation of p-cresol on EEC functions is warranted that may provide additional information on the mechanisms underlying its effects on EEC transcription.

## Data availability statement

The original contributions presented in the study are included in the article/supplementary material. Further inquiries can be directed to the corresponding author.

## Ethics statement

The animal study was reviewed and approved by Animal Experiments Inspectorate under the Danish Ministry of Food, Agriculture and Fisheries (License number - 2018-15-0201-01491).

## Author contributions

TA, FB, MG conceptualized the project. PT, AV, KT performed experiments. TA, FB, TM interpreted data. PT, TA wrote the manuscript, FB, MG critically reviewed the manuscript. All authors contributed to the article and approved the submitted version.
